# Establishment and evaluation of a carotid artery dissection model in rats

**DOI:** 10.3389/fneur.2024.1420278

**Published:** 2024-12-18

**Authors:** Shufan Zhang, Zhenxiang Han, Ying Cao, Weicheng Wu, Yuanzeng Liu, Shilin Yang, Xiaochao Feng, Chun Yu, Zhu Zhu, Qiang Dong, Xiang Han

**Affiliations:** ^1^Department of Neurology, Huashan Hospital, Fudan University, Shanghai, China; ^2^Department of Neurology and Neurological Rehabilitation, Shanghai Seventh People’s Hospital, Shanghai University of Traditional Chinese Medicine, Shanghai, China; ^3^School of Life Sciences, Fudan University, Shanghai, China; ^4^Gumei Community Health Service Center of Shanghai Minhang District, Shanghai, China; ^5^Department of Neurology, Shanghai Longhua Hospital, Shanghai, China; ^6^Department of Intensive Care Unit of West Campus, Huashan Hospital, Fudan University, Shanghai, China; ^7^Department of Neurology, Indianan University Health, Indianapolis, IN, United States

**Keywords:** carotid artery dissection, beta-aminopropionitrile (BAPN), mechanical torsion, animal model, rat

## Abstract

**Background:**

Given the lack of models for carotid artery dissections (CAD), we aim to investigate effects of beta-aminopropionitrile (BAPN) combined with physical damage on the arterial walls of rats, and to establish a high-incidence and low-mortality CAD model.

**Methods:**

Sixteen SPF SD rats (3-week-old) were divided into two groups. Group B was given 0.25% BAPN solution and group W was given water. Then we established an animal model of CAD by carotid artery torsion. One of the two carotid arteries in each rat was randomly selected for torsion. We got four groups of blood vessels following above-mentioned methods: BAPN plus torsion group (group Bt), BAPN plus non-torsion group (group Bn), water plus torsion group (group Wt), and water plus non-torsion group (group Wn). The hematoxylin and eosin (HE) staining and Verhoeff’s Van Gieson (EVG) staining were performed to observe structures of arteries. Immunofluorescence staining was used to detect structural proteins in vessels. We used triphenyltetrazolium chloride (TTC) staining and neurological function assessment to detect the infarct area of brain and neurological deficits in rats with carotid dissection to verify the validity of the rat model.

**Results:**

BAPN treatment significantly affected the weight gain of rats, but had little effect on survival during the first 5 weeks. The group Bt had the highest incidence of CAD among all groups (*p* = 0.014). HE staining of carotid artery tissue sections showed that the vascular walls were the thickest in group Bt (*p* < 0.001). EVG staining showed the arrangement of elastic fibers was the most irregular in group Bt. Immunofluorescence staining revealed that the expression of a-SMA and SM22a were decreased remarkably in group Bt (*p* < 0.001). Both motor and sensory deficits were more severe in CAD group than control group (*p* = 0.0004; *p* = 0.0036). The relative infarction volumes of CAD group rats were significantly larger than control group (*p* < 0.001).

**Conclusion:**

The animal model of CAD can be feasible to establish by mechanical torsion combined with BAPN free drinking. With this method, the animal mortality was low and the model formation rate was high. This model will enable further studies on CAD.

## Introduction

Cerebral artery dissection (CeAD) represents a major factor causing stroke in young and middle-aged adults. One of the most common types is carotid artery dissection (CAD). Studies have found that 10 to 25% of patients aged 19 to 45 years with first-episode stroke are caused by cerebral arterial dissection (1). Dissection of the arterial wall, due to a tear in the artery’s intimal layer or arterial wall bleeding, can be serious and life-threatening. The exact pathogenesis of the early stage of cerebral arterial dissection remains uncertain, because of the lack of suitable animal models (2). At present, animal models of aortic dissection and dissecting aneurysm are mainly created using mechanical methods, drug induction and gene intervention (3). As an irreversible lysyl oxidase inhibitor, BAPN can competitively bind to elastin or amino, and inhibit the cross-linking of elastin and collagen, which leads to the occurrence of dissecting aneurysms. Thus, it is a common drug to establish animal models of aortic dissection (4).

However, there is still no generally acceptable animal model of carotid artery dissection due to differences in the pathogenesis of aortic and carotid artery dissection, such as the embryonic origin of the artery, pathophysiology, etc. (5–7). The model formation rate of carotid artery dissection was low after single BAPN induction. Recently, a study has shown that using a detacher and balloon dilation could establish a CAD model successfully in swines (8), but the difficulty of interventional surgery limited the popularization of this modeling method. Therefore, the aim of this study was to investigate the effects of BAPN combined with twist and clip of carotid artery on arterial walls of rats, and to establish a high-incidence and low-mortality CAD model. A successful animal model will aid future studies focused on the development, pathogenesis and treatment for CAD.

## Methods

### Animals

Male specific-pathogen free Sprague Dawley (SD) rats (3 weeks, 60 g) provided by Shanghai Jasper Laboratory Animal Co., Ltd. and housed under specific pathogen-free (SPF) conditions, received standard chow, with free access to water. All animals underwent adaptive feeding for 3 days before further intervention. Animal experiments were performed at the Institute of Neurology, Huashan Hospital, Fudan University. The animal experiments followed the Guide for the Care and Use of Laboratory Animals, and had approval from the Institutional Animal Care and Use Committee of Huashan Hospital, Fudan University.

### Experimental design and grouping

**Figure 1:**
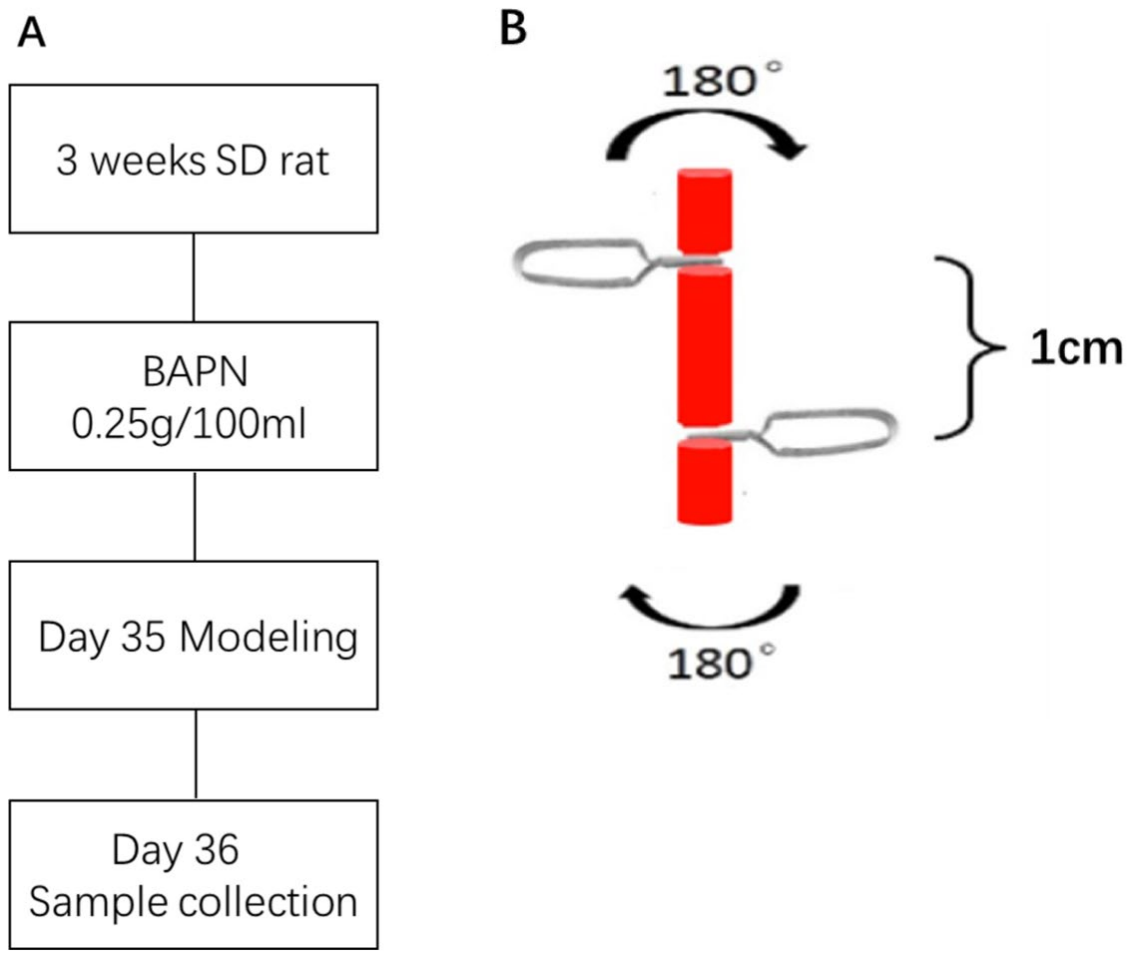
The flow diagram of model management **(A)** and the schematic of torsion process **(B)**.

Sixteen rats were randomly divided into 2 groups: BAPN group (group B), and water-control group (group W). The group B (*n* = 8) was fed by BAPN solution at a concentration of 0.25% and group W (*n* = 8) was fed by distilled water. One of the two carotid arteries in each rat was randomly selected for torsion. According to different operation, 32 vessels from 16 rats were divided into four groups: BAPN plus torsion group (group Bt), BAPN plus non-torsion group (group Bn), water plus torsion group (group Wt), and water plus non-torsion group (group Wn). The incidence of dissection was calculated at the level of each vessel. All animals had free access to food and water. Body weights were recorded every week. The flow diagram of rat management was shown in Figure 1A.

### Model establishment

- Anesthesia: Before sampling, animals were fasted for 4 h and intraperitoneally administered pentobarbital sodium (3 mL/kg body weight) for anesthesia. The experimental design and anesthesia had approval from the Animal Room Ethics Committee of Fudan University.
- Modeling: After local disinfection of the neck, the neck’s skin along the midline was longitudinally incised, and the left and right carotid arteries were carefully separated. A carotid artery was randomly selected, and two edentulous micro hemostatic clips were used to clamp the carotid artery from both sides of the head and tail in the opposite direction of the handle end (the distance between the two clips was 1 cm). Then, both clips were rotated at 180° in the opposite direction for ten minutes. Figure 1B shows the schematic of torsion process. After that both clips reverse the movement to obtain the returnability. The above operation was repeated 3 times. The other internal carotid artery was left untreated and used as a control. After the above-mentioned torsion damage, two microvascular clips were gently removed, and the skin was sutured and disinfected.

### Neurological deficits assessment

Neurological deficits of modeling rats were evaluated at 24 h before sacrificed. We used an eight-point behavioral rating scale designed by Roger et al. (9) to assess the motor function as described previously. Zero represented the normal score and the maximal deficit score was seven. In addition, we conducted hot plate test (10) to assess the sensory function. Response time for observed behavioural changes like licking the paw, lifting the claw and escaping from the hot plate was recorded. Longer response time represented more severe sensory impairment.

### Specimen collection

The rats were sacrificed using barbital sodium via intraperitoneal injection after behavioral test. Carotid arteries and brains were taken out for subsequent experiments.

### Triphenyltetrazolium chloride staining

We made coronal sections of rat brains with 2 mm thickness. The brain slices were stained with 1% triphenyltetrazolium chloride (TTC) (Sigma-Aldrich, Shanghai, China) solution for 20 min at 37 degrees. Then we used Image-Pro Plus 6.0 software to calculate the ratio of the ischemic area to the ipsilateral hemisphere area (I/H ratio).

### Hematoxylin and eosin staining and Verhoeff’s Van Gieson staining

Carotid artery tissue specimens from normal animals and carotid artery dissection model animals were collected. Upon fixation with 4% formalin for 24 h, the tissue specimens underwent dehydration with graded ethanol and N-butanol. Then the specimens were embedded in paraffin at 60°C, followed by 5-μm serial sectioning.

Before staining, the sections were dewaxed with xylene and graded ethanol. Specifically, the sections were soaked twice in xylene for 10 min and then soaked in graded ethanol for 5 min each time.

For H&E staining, first we stained the tissue sections with hematoxylin for 3–5 min. After incubation with acid and ammonia solutions for 40 s each, the samples were dyed with eosin for 2 min. Then the slices were dehydrated in graded ethanol and cleared in 100% ethanol and xylene. An inverted microscope (Olympus, Tokyo, Japan) was utilized for analysis.

In EVG staining, the tissue sections were incubated with EVG solution (hematoxylin, iodine solution and ferric chloride at 5:2:2) for 20 min followed by washing with deionised water. Then VG solution (saturated picric acid and Fuchsin solution at 9:1) was used to stained the sections for 5 min followed by washing. The ferric chloride differentiation solution was dyed for background until a grey white background was obtained. Finally, the slices were dehydrated in graded ethanol and cleared in 100% ethanol and xylene. An inverted microscope (Olympus, Tokyo, Japan) was utilized for analysis.

### Immunofluorescence staining

Before staining, tissue sections were dewaxed with xylene and graded ethanol. Specifically, the sections were soaked twice in xylene for 10 min and then soaked in graded ethanol for 5 min each time. Then we used citric acid buffer (PH6.0) to retrieve antigen in microwave. As for staining stage, the samples were blocked by 3% BSA, permeabilized by TBS with 0.3% Triton X-100 and incubated by antibodies. The primary antibodies binding to smooth muscle alpha actin (a-SMA) (Affinity, AF1032) and smooth muscle 22 alpha (SM22a) (Proteintech, 10493-1-AP) should be incubated overnight at 4°C. The secondary antibody (Abcam, ab175470) should be incubated at 37°C for 50 min. DAPI in anti-fading sealant (Servicebio Technology Co., Ltd., Wuhan, China) was used to counterstain nuclei. Finally, samples were observed via fluorescence microscope (Olympus, Tokyo, Japan).

### Measurement of vessel wall thickness

The thickness of the carotid artery wall was defined as the width of the wall at its thickest point. The thickness was measured by Image-Pro Plus 6.0 software in HE staining sections.

### Statistical analysis

All normally distributed data were presented as mean ± standard deviation, while categorical variables were presented as proportion of total. SPSS 22.0 software was used for Student’s *t*-test, chi-square test and Fisher’s exact probability tests. GraphPad 8.0 software was used for plotting data. Image-Pro Plus 6.0 software was utilized for image analysis and determination of vascular parameters. All *in vitro* experiments were performed at least three times. *p* < 0.05 reflected statistical significance.

## Results

### Effects of BAPN on body weight and survival

**Figure 2:**
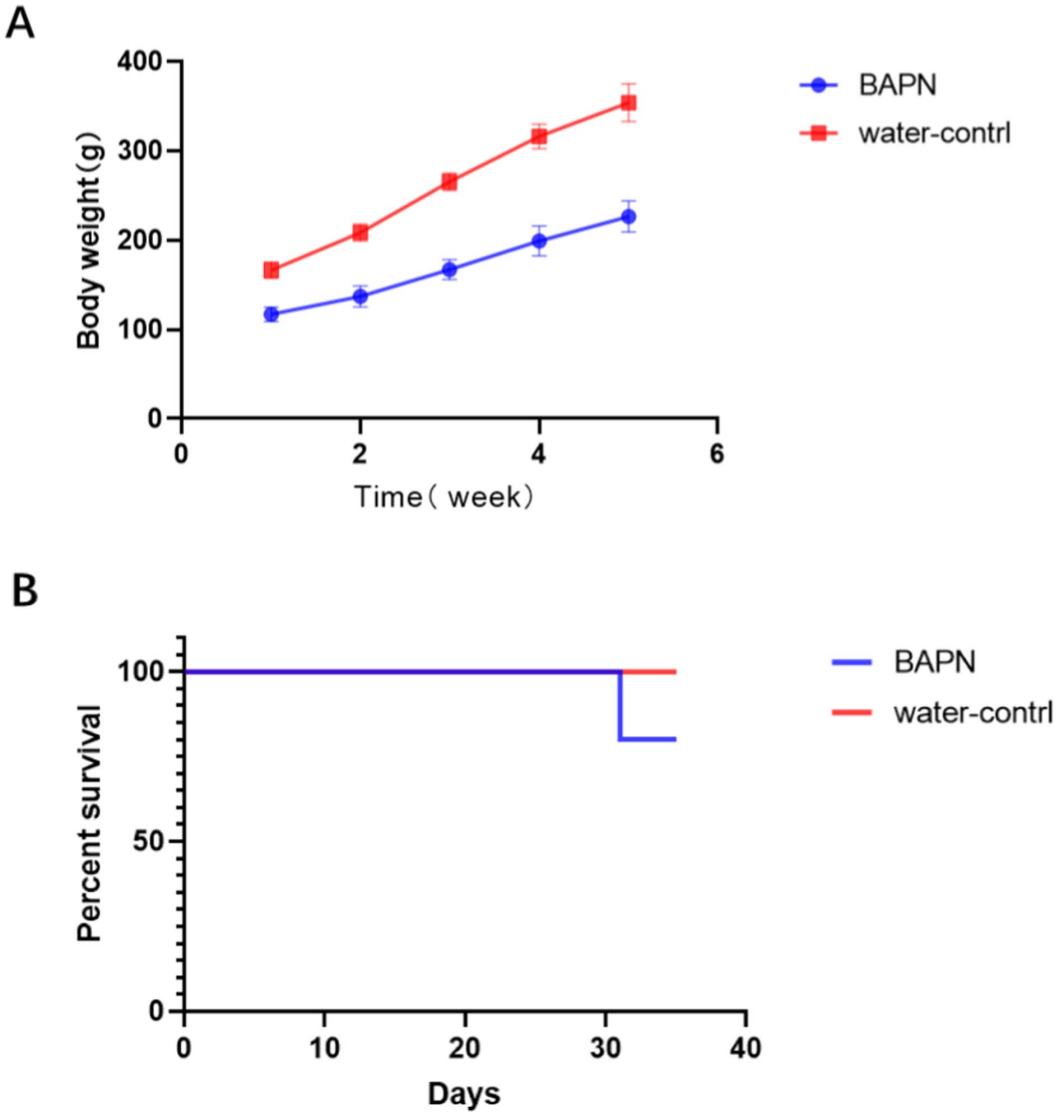
Changes in body weight **(A)** and survival curves of SD rats **(B)** during 5 weeks.

With the increase of BAPN intake, the rat body weight showed a decreasing trend (Figure 2A). Only one rat in the BAPN group died by the fifth week (Figure 2B).

### Modeling efficiency

An animal model of carotid artery dissection was established with mechanical clip rotation as previously described (Figures 1A,B). After modeling, the rate of CAD occurrence was the highest in the group Bt at 71.4%, followed by the 28.6, 25%, and 0 in group Bn, group Wt and group Wn, respectively (Table 1). Effects of different treatments on the incidence of CAD were shown in Table 2. The incidence of dissection in the BAPN group and BAPN combined torsion group were significantly higher than in other groups (*p* = 0.046; *p* = 0.014). There was no significant difference in the rate of CAD between the torsion group and other groups (*p* = 0.109). In order to explore the separate effects of BAPN or torsion damage, we chose the BAPN group without torsion intervention or the torsion group without BAPN intervention as single-BAPN group or single-torsion group, respectively. Finally, the incidence of CAD in both single-BAPN group and single-torsion group was not significantly different from the other groups (both *p* = 1).

**Table 1:** Incidence of carotid artery dissection in each group at 5 weeks of different treatments.

		Dissection	No-dissection
BAPN	Torsion (*n* = 7)	5 (71.4%)	2 (28.6%)
	No-torsion (*n* = 7)	2 (28.6%)	5 (71.4%)
Water-control	Torsion (*n* = 8)	2 (25%)	6 (75%)
	No-torsion (*n* = 8)	0 (0)	8 (100%)

**Table 2:** Incidence of carotid artery dissection between groups of different treatments.

Group	Dissection	No-dissection	*p*
BAPN (*n* = 14)	7 (50%)	7	0.046
Water-control (*n* = 16)	2	14	
Torsion (*n* = 15)	7	8	0.109
No-torsion (*n* = 15)	2	13	
BAPN-torsion (*n* = 7)	5 (71.4%)	2	0.014
Rests (*n* = 23)	4	19	
Control (*n* = 8)	0	8	0.067
Rests (*n* = 22)	9	13	
Single-BAPN (*n* = 7)	2	5	1
Rests (*n* = 23)	7	16	
Single-torsion (*n* = 8)	2	6	1
Rests (*n* = 22)	7	15	

### Large artery lesions

#### Gross anatomy of the carotid artery

**Figure 3:**
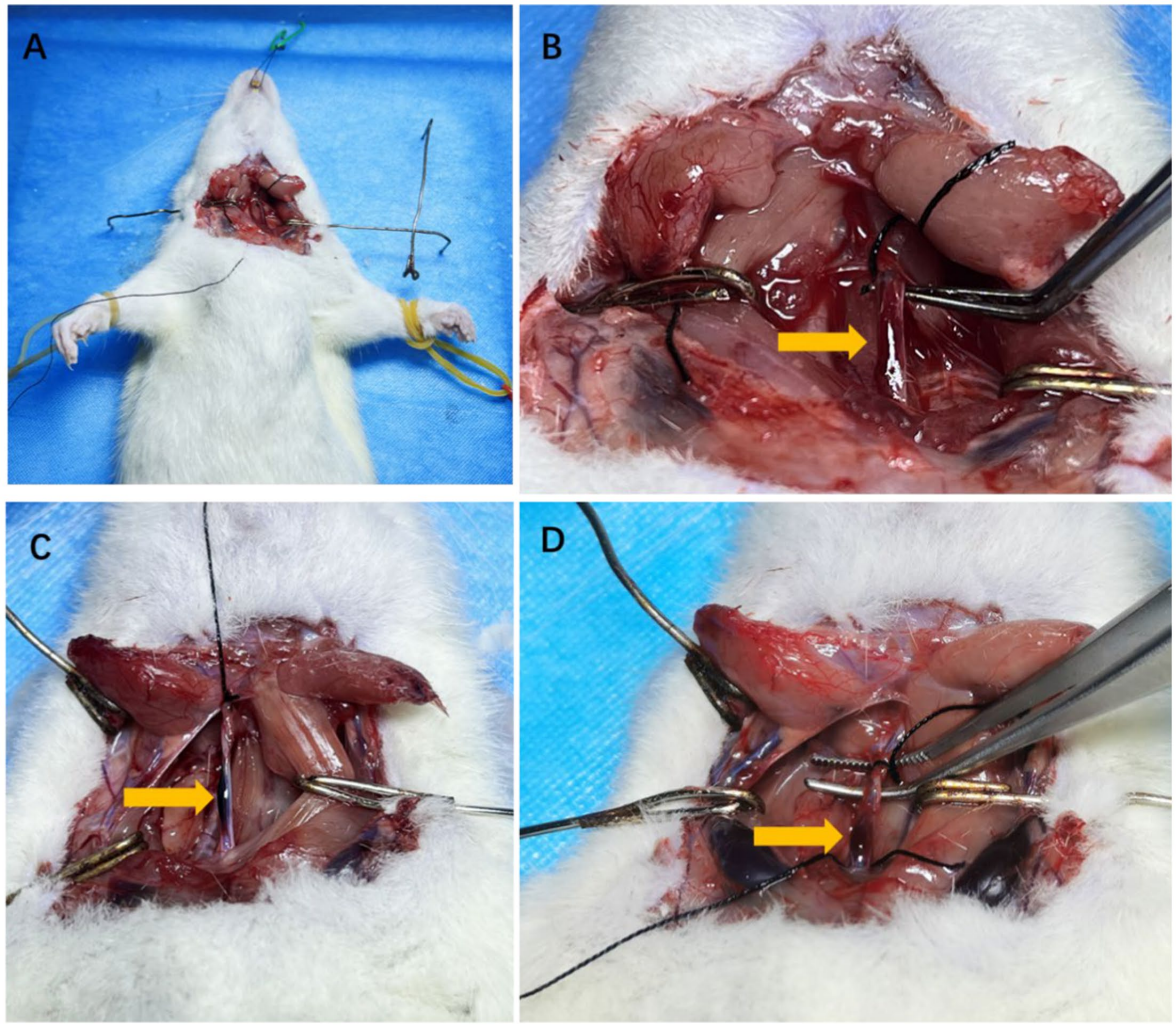
The anatomical pictures of model rats and carotid artery dissection sample. The example of surgical incision was shown in **A**. The isolated carotid arteries of rats and the wall hematoma caused by carotid artery dissection after modeling were shown by arrows in **B–D**.

In rats with CAD, intramural hematomas were observed in carotid arteries. Hematomas were located at the twisting point between the two clips (Figure 3).

#### Vascular pathological structure

**Figure 4:**
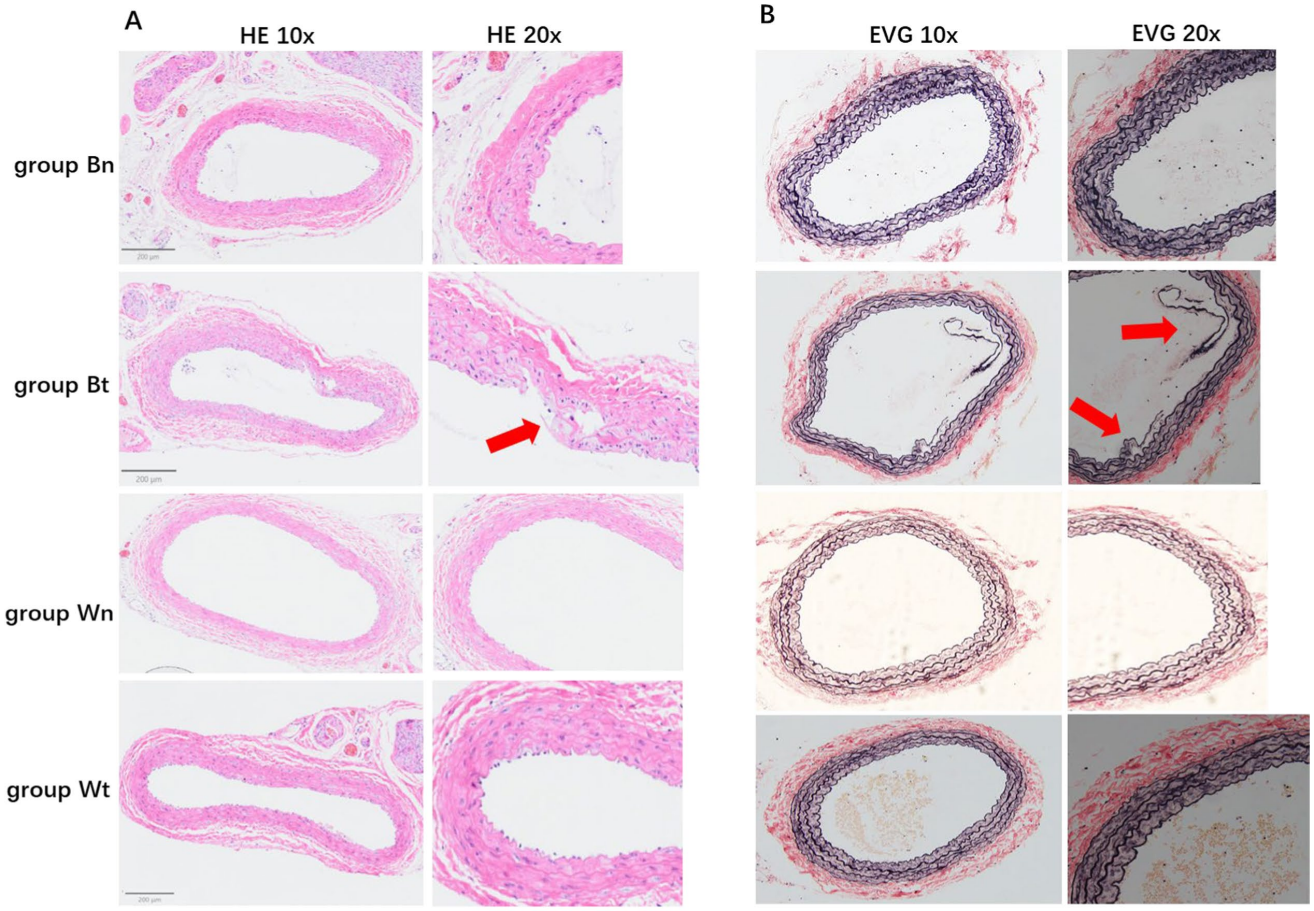
HE staining and EVG staining for each group. The HE staining of tissue sections in different groups were shown in **A**. The EVG staining of tissue sections in different groups were shown in **B**. The arrows showed the carotid artery dissections in different staining methods.

**Figure 5:**
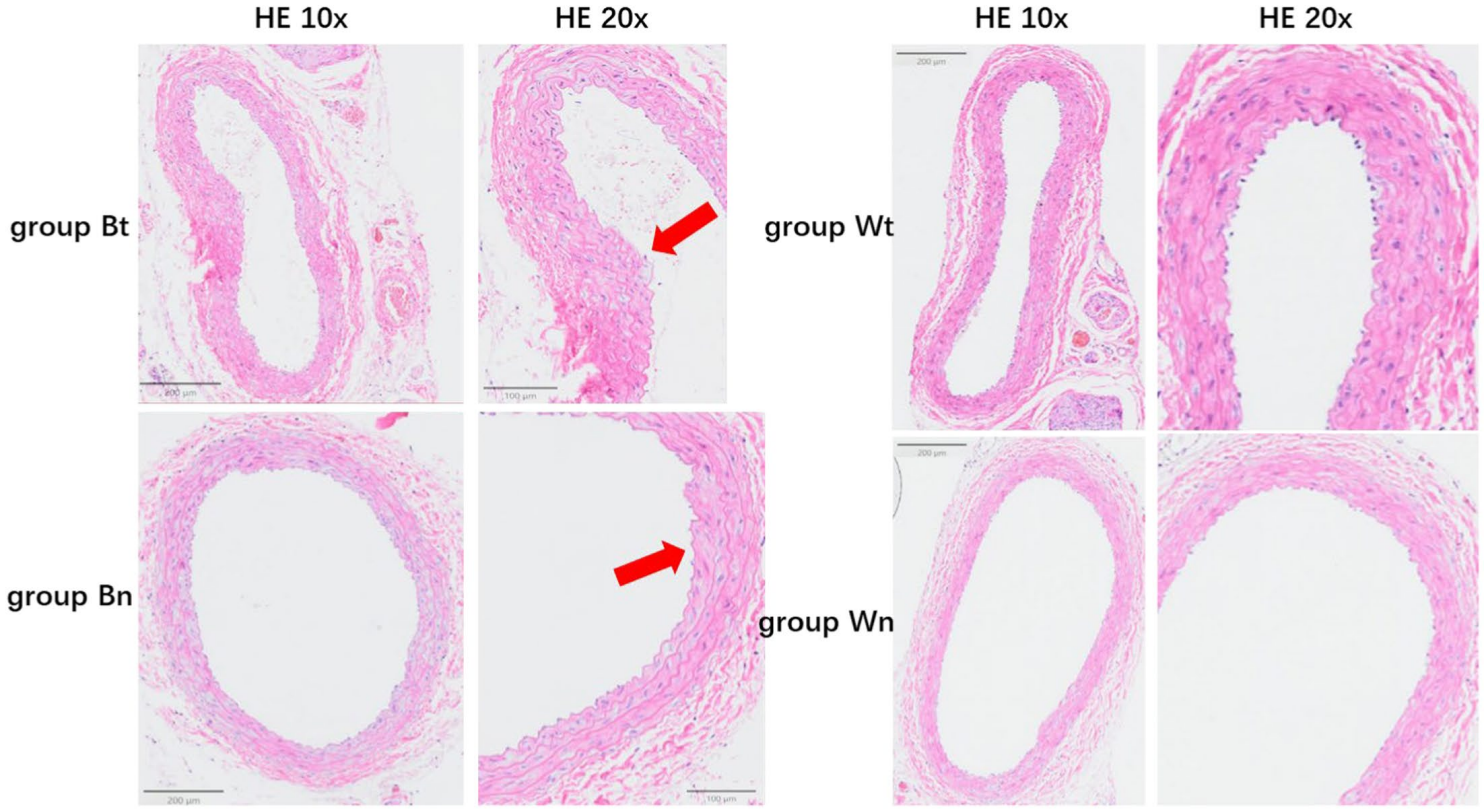
The average thickness of the wall of the carotid artery in each group. The arrows showed the thickened vessel walls in group Bt and group Bn.

**Figure 6:**
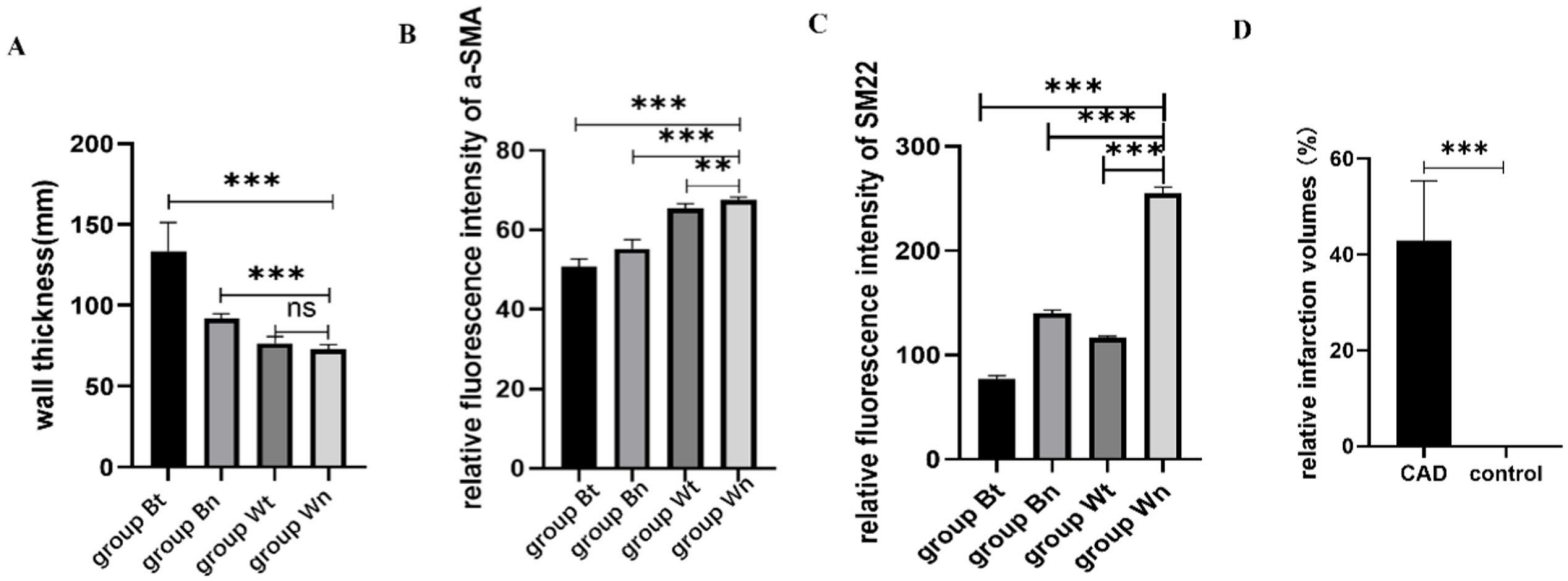
The statistical results of average wall thickness, expression levels of contractile proteins and relative infarction volumes. The average thickness of the walls in group Bt and group Bn were significantly thicker than that in group Wn. There was no significant difference between group Wt and group Wn in average wall thickness **(A)**. The expression of a-SMA protein in group Bt, group Bn and group Wt were significantly decreased compared with group Wn. The decrease in group Bn and group Wt were not as obvious as that in group Bt **(B)**. The expression of SM22a protein in group Bt, group Bn and group Wt were significantly decreased compared with group Wn. The decrease in group Bn and group Wt were not as obvious as that in group Bt **(C)**. The relative infarction volumes of CAD group rats were significantly larger than control group **(D)**.

HE staining showed that compared with group Wn, the number of nuclei in the blood vessel wall increased significantly and the arrangement of cells in the wall became more irregular both in group Bt and group Bn. However, the number of nuclei and arrangement of cells in group Wt were similar to that in group Wn (Figure 4A). EVG elastic fiber staining showed that compared with group Wn, the elastic fibers were disorganized and had multiple breaks in group Bt and group Bn. However, the morphology of elastic fibers in group Wt were similar to that in group Wn (Figure 4B). The average thickness of carotid artery walls in group Bt and group Bn was significantly thicker than that in group Wn (Figures 5, 6A, both *p* < 0.001). There was no significant difference between group Wt and group Wn in average wall thickness (Figures 5, 6A, *p* = 0.054).

### Contractile protein of vascular smooth muscle cell

**Figure 7:**
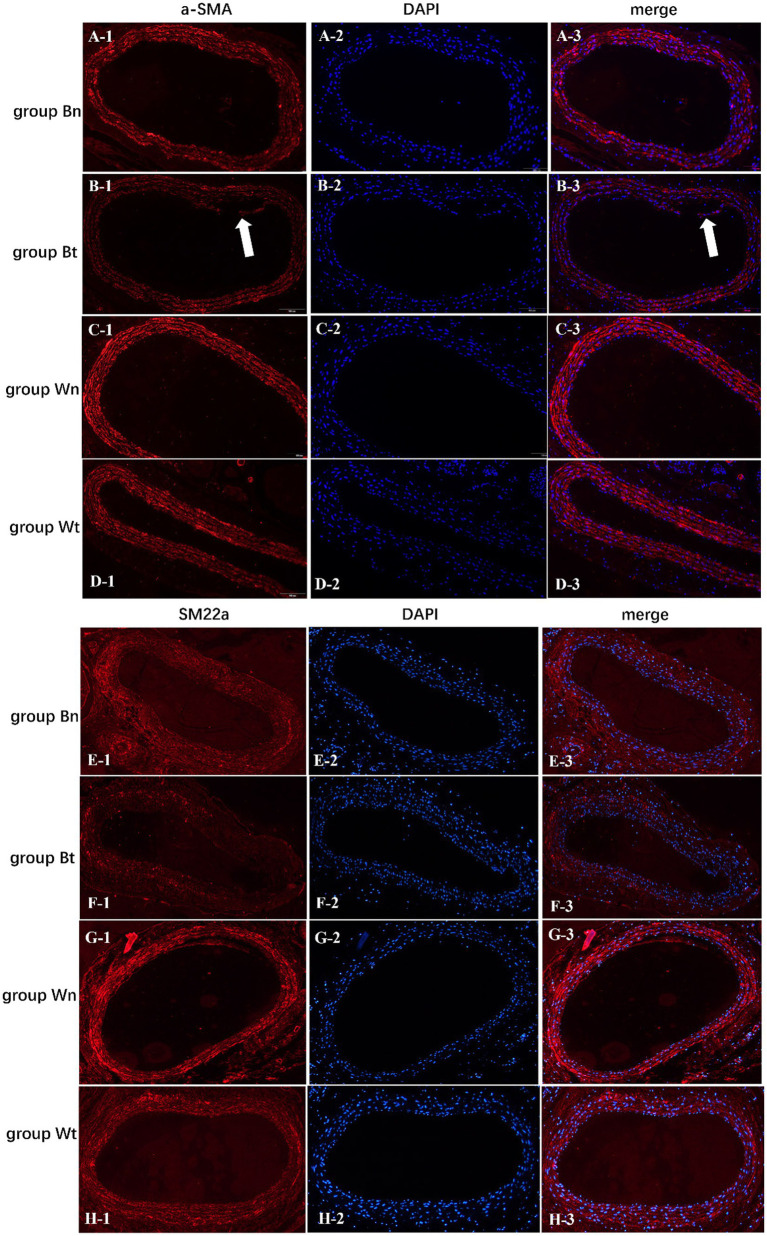
The expression levels of a-SMA and SM22a contractile proteins in vascular smooth muscle cells of each group. The arrows showed the carotid artery dissections in group Bt.

To investigate the effect of the arterial dissection on the contractile protein of vascular smooth muscle cell (VSMC), we detected a-SMA and SM22a levels in walls of dissected vessels versus normal vessels by immunofluorescence staining. The expression of a-SMA and SM22a proteins in group Bt, group Bn and group Wt was decreased significantly compared with group Wn (a-SMA: *p* < 0.001; *p* < 0.001; *p* = 0.0011, SM22a: both *p* < 0.001). However, a-SMA and SM22a levels in group Bn and group Wt were similar (Figures 6B,C, 7A–H).

### Neurological function in CAD model

After completing the modeling process, we compared the neurological function between the CAD group and the control group to verify the neurological deficits of the rat model. The CAD group was composed of 5 rats with carotid artery dissection from Bt group. The control group was composed of 5 rats that were randomly selected from Wn group. Eight-point Roger scale and hot plate test were used to evaluated the motor and sensory function, respectively. The average Roger score of CAD group was significantly higher than that of control group (3.4 ± 1.3 vs. 0, *p* = 0.0004). Average response time of CAD group was significantly longer than that of control group (6.6 ± 1.3 s vs. 3.8 ± 0.83 s, *p* = 0.0036) (Table 3).

**Table 3:** Roger’s score and time of hot plate test between CAD group and control group.

Group	CAD	Control	*p*
Roger score	3.4 ± 1.3	0	0.0004
Time (s)	6.6 ± 1.3	3.8 ± 0.83	0.0036

### The volume of the infarct area in successful model with carotid dissection

**Figure 8:**
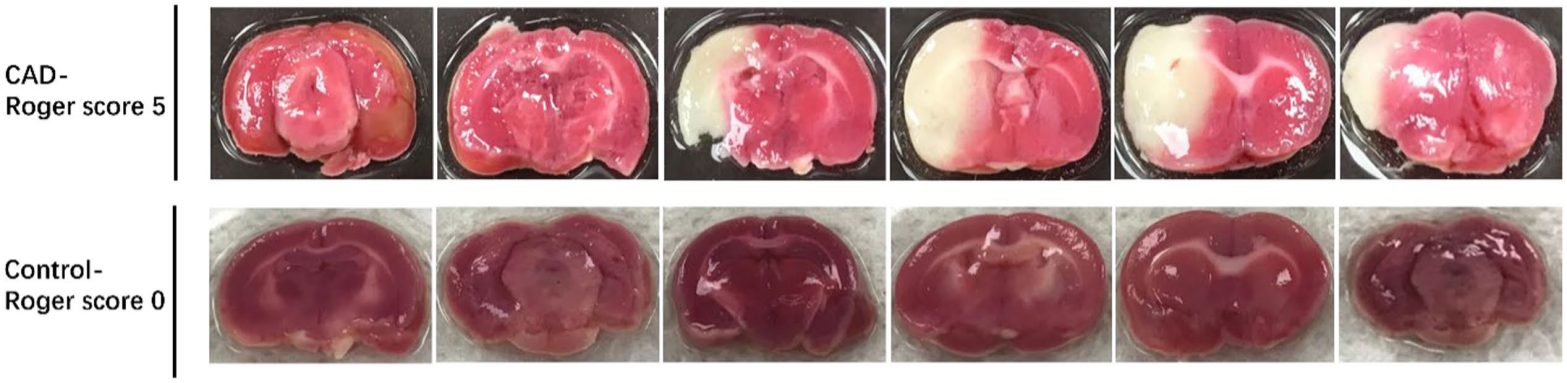
The volumes of the infarct area between CAD group with the most severe neurological deficits and the control group.

We compared the volume of the infarct area between the CAD group and the control group to verify the validity of the rat model. The CAD group was composed of 5 rats with carotid artery dissection from Bt group. The control group was composed of 5 rats that were randomly selected from Wn group. The relative infarction volumes of CAD group rats were significantly larger than control group (46.87% ± 12.47% vs. 0, *p* < 0.001) (Figure 6D). Figure 8 showed a comparison of the brain sections of the rat with the most severe neurological deficits in the CAD group versus those in the control group.

## Discussion

CAD represents an important cause of stroke in young and middle-aged adults. However, due to the lack of suitable animal models, researches on the pathogenesis of CAD remains scarce (11). Therefore, it is meaningful to establish a high-incidence and low-mortality CAD model.

Previous studies related to aortic dissection have focused on genetic susceptibility and pathogenesis (12). There are many animal models of aortic dissection, including mice, rats, rabbits, dogs, and pigs. Methods of model establishment mainly include surgical intervention, drug induction and gene knockout. Among them, balloon dilatation and local trypsin injection were mainly used in large animals, while drug induction was mainly used in small animals. Balloon dilatation and local trypsin injection were limited due to difficult operation, but Ang II and BAPN were widely used to induce aortic dissections or aneurysms. Drug designs varied in different studies which included the administration of BAPN alone, the combination of BAPN and AngII, and the simultaneous administration of BAPN and Ang II (13–15).

As an irreversible lysyl oxidase inhibitor, BAPN can competitively bind to elastin or amino and alkyl groups, thereby inhibiting the cross-linking of elastin to collagen (16). Treatment with BAPN resulted in thickening of the tunica media of large arteries, irregularities in the arrangement of elastin and collagen fibers, and necrosis. These pathological conditions are consistent with lesions of human arterial dissection. Previous studies indicated that high BAPN concentration was more likely to lead to aneurysm formation and increased mortality, so we feeded rats with a lower concentration at 0.25% (16–18). However, the pathogenesis of carotid artery dissection is not exactly the same as that of aorta, especially in terms of mechanical injury, such as massage, yoga, etc., which is considered to be inseparable from the occurrence of carotid artery dissection. In this study, we made a new attempt to choose 0.25% BAPN solution combined with carotid artery torsion to establish CAD models rather than the classical method in previous aortic dissections models. The animal mortality was low and the model formation rate was high in our study. Moreover, compared with the control group, we also observed that arterial walls of the CAD are significantly thickened, the number of VSMCs is increased, and the arrangement of elastic fibers is disordered.

We chose the three-week-old SD rats for modeling because at this rapid growth stage, BAPN could inhibit the generation of the ECM in large arteries, which then led to formation of aneurysms or aortic dissections easily (17–19).

The destruction of structural and functional integrity of the vessel wall are essential pathological changes in arterial dissection (20). The a-SMA and SM22a are generally referred to as “contractile” isoforms mostly expressed in vascular smooth muscle (21, 22). Previous studies suggested that mutations in ACTA2, the gene encoding a-SMA, are the most frequent cause of non-syndromic, heritable thoracic aortic aneurysms and dissection (23). As VSMC-specific contractile proteins, a-SMA and SM22a are characterized by reduction in both thoracic and abdominal aneurysmal aortic tissues (24). Therefore, a-SMA and SM22a were commonly used as markers to reflect the function of VSMC. In this study, significantly lower a-SMA and SM22a levels were observed in the carotid artery walls of successfully modeled CAD rats compared to controls.

In addition to the vessel wall lesions, we also detect the infarct area of brain and neurological deficits in rats with carotid dissection to verify the validity of the rat model. Rats with severe carotid artery dissection were noticed to have larger infarcts in the areas of the brain which were supplied by the carotid artery. Accordingly, the neurological deficit scores of the rats were also high. These results were consistent with clinical manifestations of carotid dissection in humans. In fact, not all CAD patients develop severe clinical symptoms (25, 26).

During our modeling process, changes of each factor influencing the pathological mechanisms of carotid dissection were assessed. We found that the incidence of dissection was low in both group Bn and group Wt, but when the two conditions were combined, the incidence in group Bt was significantly increased. These results were consistent with the pathophysiology of carotid dissection in humans, that is, external injuries, yoga, and massage are prone to cause carotid artery dissection (27, 28), and also reflected the rationality of our methods.

This study has some limitations. First, the sample size is small, and the stability of the model requires multiple experiments with larger sample sizes. In addition, this animal model could not simulate carotid arterial dissection caused by specific mechanisms, such as genetic mutations. However, an animal model that can mimic the pathophysiological mechanism of carotid artery dissection remains lacking to date, so our approach might be used in future studies as an innovative attempt.

## Data Availability

The original contributions presented in the study are included in the article/supplementary material, further inquiries can be directed to the corresponding authors.
